# The use of moderate hypothermia during cardiac surgery is associated with repression of tumour necrosis factor-α via inhibition of activating protein-1: an experimental study

**DOI:** 10.1186/cc4886

**Published:** 2006-04-07

**Authors:** Ma Qing, Michael Wöltje, Kathrin Schumacher, Magdalena Sokalska, Jaime F Vazquez-Jimenez, Ralf Minkenberg, Marie-Christine Seghaye

**Affiliations:** 1 Department of Pediatric Cardiology, Aachen University Hospital, Aachen, Germany; 2 Interdisciplinary Center for Clinical Research, BIOMAT, Aachen University Hospital, Aachen, Germany; 3 Department of Pediatric Cardiac Surgery, Aachen University Hospital, Aachen, Germany; 4 Repges and Co. Institute for Medical Statistics, Aachen, Germany

## Abstract

**Introduction:**

The use of moderate hypothermia during experimental cardiac surgery is associated with decreased expression of tumour necrosis factor (TNF)-α in myocardium and with myocardial protection. In order to identify the cellular mechanisms that lead to that repression, we investigated the effect of hypothermia during cardiac surgery on both main signalling pathways involved in systemic inflammation, namely the nuclear factor-κB (NF-κB) and activating protein-1 pathways.

**Method:**

Twelve female pigs were randomly subjected to standardized cardiopulmonary bypass with moderate hypothermia or normothermia (temperature 28°C and 37°C, respectively; six pigs in each group). Myocardial probes were sampled from the right ventricle before, during and 6 hours after bypass. We detected mRNA encoding TNF-α by competitive RT-PCR and measured protein levels of TNF-α, inducible nitric oxide synthase and cyclo-oxygenase-2 by Western blotting. Finally, we assessed the activation of NF-κB and activating protein-1, as well as phosphorylation of p38 mitogen-activated protein kinase by electrophoretic mobility shift assay with super shift and/or Western blot.

**Results:**

During and after cardiac surgery, animals subjected to hypothermia exhibited lower expression of TNF-α and cyclo-oxygenase-2 but not of inducible nitric oxide synthase. This was associated with lower activation of p38 mitogen-activated protein kinase and of its downstream effector activating protein-1 in hypothermic animals. In contrast, NF-κB activity was no different between groups.

**Conclusion:**

These findings indicate that the repression of TNF-α associated with moderate hypothermia during cardiac surgery is associated with inhibition of the mitogen-activated protein kinase p38/activating protein-1 pathway and not with inhibition of NF-κB. The use of moderate hypothermia during cardiac surgery may mitigate the perioperative systemic inflammatory response and its complications.

## Introduction

Myocardial damage is an important complication of cardiac surgery involving cardiopulmonary bypass (CPB) [1]. Synthesis of tumour necrosis factor (TNF)-α in the myocardium is thought to play a central role in its pathophysiology [2,3]. Indeed, there is a large body of evidence that, in experimental models, over-expression of TNF-α in the myocardium is related to adverse cardiac effects such as postinfarct remodelling and ventricular dilatation [4], transition from hypertrophic to dilated cardiomyopathy due to apoptosis [5] and impaired postischaemic functional recovery [6]. Additionally, local administration of soluble TNF-α receptor-1 gene reduced infarct size in a model of ischaemia/reperfusion injury [7]. In a study conducted in a neonatal model of ischaemia of the hypertrophied left ventricle, inhibition of the biological activity of TNF-α significantly improved postischaemic contractile function, myocardial energetics and intracellular calcium handing [8]. In humans there is a clear relationship between TNF-α expression in the myocardium and the severity of dilated cardiomyopathy [9,10].

The nuclear factor-κB (NF-κB) family of nuclear transcription factors is critical for the synthesis of TNF-α and for TNF-α induced secondary mediators of inflammation, such as inducible nitric oxide synthase (iNOS) and cyclo-oxygenase (COX)-2 [11]. Inflammatory stimuli lead to activation of NF-κB by inducing the phosphorylation of its inhibitory protein IκB, allowing its translocation into the nucleus [11-13]. Activating protein (AP)-1 is another major transcription factor for many inflammatory mediators, including TNF-α. It comprises a family of related transcription factors, consisting of heterodimers and homodimers of Jun, Fos and activating transcription factor [14]. AP-1 activity is regulated through interactions with extracellular and intracellular signals including p38 mitogen-activated protein kinase (MAPK), with phosphorylation of activating transcription factor-2 [14], which leads to expression of TNF-α [15].

Upon activation of NF-κB and AP-1 by inflammatory stimuli, expression of inflammatory genes such as that encoding TNF-α and of proinflammatory enzymes such as iNOS and COX-2 takes place. In the myocardium, activation of NF-κB, p38 MAPK and AP-1 causes myocardial cell damage resulting from TNF-α production [16-18] and it contributes to perfusion maldistribution and to myocardial damage by nitric oxide and eicosanoids, caused by the activity of iNOS and COX-2, respectively [19].

Our previous experimental studies showed that moderate hypothermia during cardiac surgery involving CPB is related to repression of TNF-α, and that this is related to increased synthesis of interleukin-10 in myocardium [2,20]. In the present study we investigated the signalling pathways involved in this repression and found that the use of moderate hypothermia is associated with the inhibition of the p38-MAPK/AP-1 pathway but not with inhibition of the NF-κB pathway.

## Materials and methods

### Animals

The study was approved by the supervising state agency for animal experiments. Twelve stress-resistant female pigs (deutsche Landrasse) weighing 40.3 ± 1.4 kg (mean ± standard deviation) were included. The animals were housed in the institute for animal experimentation located in our university hospital for at least 8 days before experiments were begun; this was to guarantee quiet care before scheduled cardiac surgery. After clinical veterinary examination was conducted, which confirmed that the animals were in good health, the pigs were randomly assigned to a temperature group during CPB (six pigs in each group): moderate hypothermia (28°C) and normothermia (37°C). Core temperature was monitored using an oesophageal probe (probe 1651; Datex-Ohmeda Division, Instrumentarium Corp., Helsinki, Finland).

### Surgical procedure

General anaesthesia, and operative and CPB technique were as previously described [2]. Briefly, following sternotomy, cephotiam (50 mg/kg intravenously) and heparin were administered, and both caval veins, the aorta and the left atrium were cannulated and total CPB instituted for 120 minutes in all animals. This included 30 minutes perfusion during which animals subjected to moderate hypothermia during the operation were cooled down to 28°C; 60 minutes of perfusion during which the aorta was cross-clamped and right atriotomy performed; and 30 minutes of perfusion during which pigs undergoing hypothermic CPB were rewarmed to 37°C. In animals subjected to normothermia during the operation, perfusion was performed at 37°C for the 120 minutes. CPB was conducted with a flow index of 2.7 l/(minute. m^2 ^body surface area) in all animals, with a target mean systemic arterial pressure of 60 mmHg. Immediately after aortic cross-clamping, cardioplegia was achieved by a single injection of cold (4°C) cardioplegic solution (Bretschneider solution; 30 ml/kg) into the aortic root. Additional topical cooling of the myocardium was performed by application of 500 ml cold (4°C) saline solution. Myocardial temperature during CPB was monitored using a needle probe placed into the ventricular septum (Temperature Sensing Catheter; Medtronic Hemotec. Inc, Englewood, CO, USA). At the end of CPB, anticoagulation was reversed with protamine, mediastinal drains were placed and the chest was closed.

### Postoperative care

The lungs of the pigs were mechanically ventilated until the end of the experiment. Postoperative monitoring included continuous registration of heart rate and rhythm, mean arterial blood pressure, left atrial pressure, oesophageal temperature and urine output, and measurement of arterial lactate levels and blood gases. Animals received dopamine and Ringer's lactate to optimize hemodynamics. Six hours after sternal closure the pigs were killed by phenobarbital overdose.

### Tissue sampling

Samples for RT-PCR, Western blot and electrophoretic mobility shift assay were rapidly excised from the apex of the right ventricle before CPB, before aortic cross-clamping, before opening the aorta and immediately after death. Samples were snap frozen in liquid nitrogen and stored at -70°C.

### Reverse transcriptase polymerase chain reaction

Total RNA was extracted using Rneasy Mini Kit (QIAGEN Inc., Hilden, Germany). RNA (3 μg) was reverse transcribed to cDNA using random hexamers. Using specific porcine primers for TNF-α and β-actin, cDNA products were coamplified by PCR as previously reported [2]. The PCR products were subjected to electrophoresis in 1.8% agarose gel, stained with ethidium bromide and photographed. The predicted lengths of amplification products for TNF-α and β-actin were 372 and 233 base pairs, respectively. Results are presented as ratio of band intensities of the mRNA of TNF-α to the corresponding β-actin mRNA (Quantity One^® ^Quantification software 4.1; Bio-Rad: BioRad Laboratories, Inc., Hercules, CA, USA).

### Western blot

The samples (100 μg) were treated with SDS-PAGE sample buffer, followed by heating, and were then subjected to 8% or 12% gels. Western blots were performed with antibodies against polyclonal goat anti-human TNF-α (DPC Biermann GmbH, Bad Nauheim, Germany), monoclonal mouse anti-human iNOS (BD Transduction Laboratories, Heidelberg, Germany), polyclonal rabbit anti-human COX-2 (Alexis Deutschland GmbH, Grünberg, Germany), monoclonal mouse anti-human phospho-IκB-α (Ser32/36), polyclonal rabbit anti-human phospho-p38 MAPK and p38 MAPK (Cell Signalling Technology, Inc., Frankfurt am Main, Germany), and polyclonal goat anti-human actin (DAKO, Glostrup, Denmark). The bands were detected using a chemiluminescent system. Restaining with actin antibody ensured equal loading. Band intensities of TNF-α, iNOS, COX-2 and phospho-IκB-α were normalized to that of actin, and those of phospho-p38 MAPK were normalized to that of p-38 MAPK (Quantity One^® ^Quantification software 4.1; Bio-Rad).

For measurement of the concentrations of phospho-c-Jun and c-Jun, nuclear extracts (20 μg) were treated with SDS-PAGE sample buffer, followed by heating, and were then subjected to 10% gels. Western blots were performed with antibodies against monoclonal mouse anti-human phospho-c-Jun and polyclonal rabbit anti-human c-Jun (both Santa Cruz Biotechnology, Heidelberg, Germany) and polyclonal goat anti-human actin (DAKO). The bands were detected using a chemiluminescent system. Restaining with actin antibody ensured equal loading. Band intensities of phospho-c-Jun and c-Jun were normalized to that of actin (Quantity One^® ^Quantification software 4.1; Bio-Rad).

### Electrophoretic mobility shift assay

Nuclear extracts were prepared as previously described [21]. Protein concentrations were determined using a Bio-Rad protein assay. The electrophoretic mobility shift assay was performed using a double-stranded ^32^P-labelled mutated sis-inducible element oligonucleotide from NF-κB (5'-AGT TGA GGG GAC TTT CC-3') and using a double-stranded ^32^P-labelled consensus oligonucleotide from AP-1 (5'-CGC TTG ATG ACT CAG CCG GAA-3'; both MWG-Biotech AG, Ebersberg, Germany). For supershift assays, nuclear extracts were incubated with antibodies against NF-κB p50 and NF-κB p65 subunits and AP-1 c-Jun subunit (all polyclonal rabbit anti-human; Santa Cruz Biotechnology) for 30 minutes at room temperature before addition of the radiolabelled probe. The protein/DNA complexes were separated on a 6% polyacrylamide gel containing 7.5% glycerol in 0.25-fold tuberculin bacillary emulsion (20 mmol/l Tris, 20 mmol/l boric acid, 0.5 mmol/l EDTA) at 210 V for 3 hours. Gels were dried and autoradiographed. Positive controls for AP-1 were performed on human HepG2 stimulated by TNF-α 10 ng/ml for 4 hours.

### Statistical analysis

Results are expressed as mean ± standard deviation. Data were analyzed by analysis of variance with adjustment of *P *values by the *t *test. Differences between time points within groups were calculated using paired *t* tests using the Statistical Package for Social Sciences (SPSS; SPSS Software GmbH, Munich, Germany).

## Results

### Oesophageal and myocardial temperatures

Oesophageal temperature during and after CPB was significantly lower in animals operated on under hypothermia than in the other animals. In contrast, myocardial temperature during CPB did not differ significantly between groups, with the exception of that measured before cross-clamping of the aorta (Table 1).

### Haemodynamics and lactate levels

Heart rate, mean arterial and left and right atrial pressures, cathecolamine support and urine output were not significantly different between groups (data not shown). Lactate levels were lower at the end of CPB in animals operated on under moderate hypothermia than in the other animals (4.8 ± 0.3 mg/ml versus 6.9 ± 0.3 mg/ml; *P *< 0.002).

### Myocardial expression of tumour necrosis factor-α

**Figure 1:**
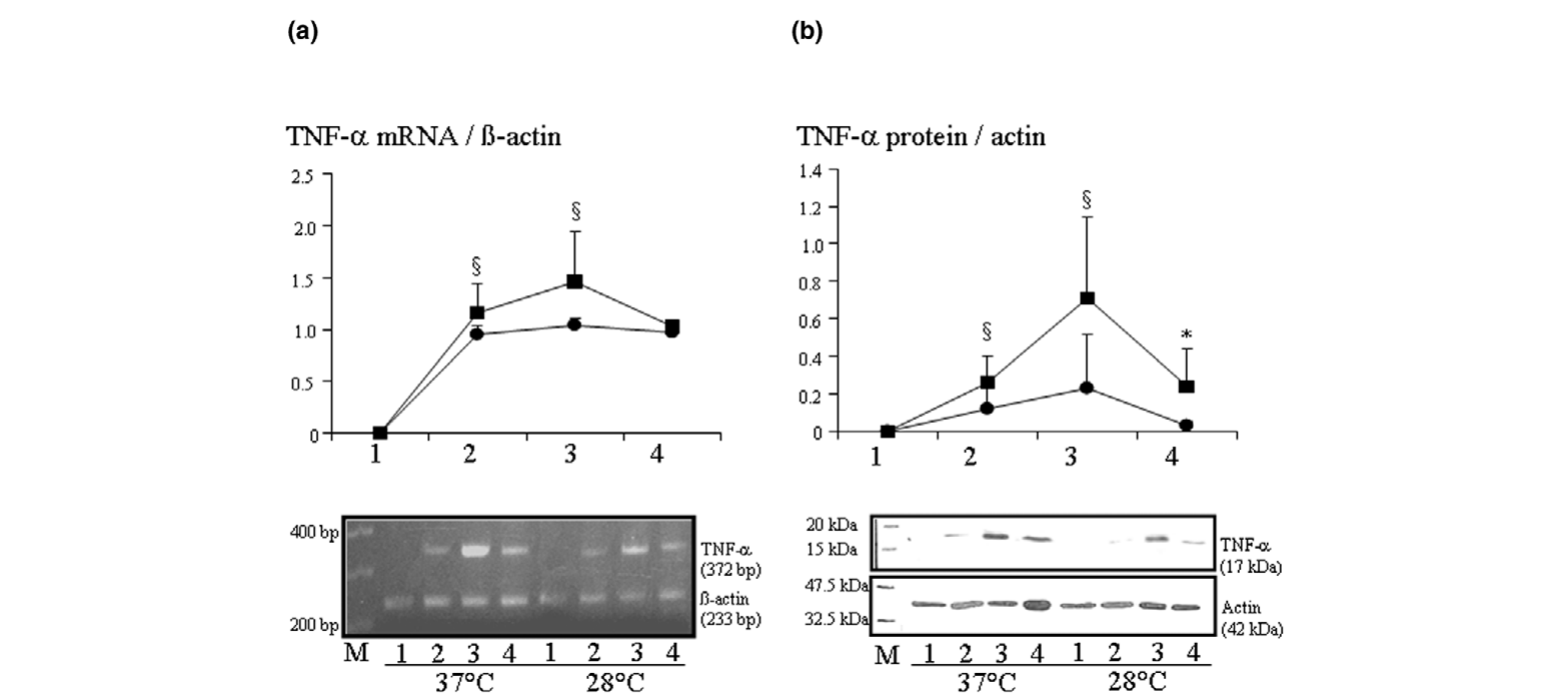
TNF-α mRNA expression and TNF-α concentration: hypothermia versus normothermia. Shown are the expression of TNF-α mRNA and TNF-α concentrations in pigs operated on under moderate hypothermia (28°C; circles) or normothermia (37°C; squares). The time points of evaluation were as follows: 1 = before CPB; 2 = before aortic cross clamping; 3 = before removal of aortic clamp; and 4 = 6 hours after CPB. **(a) **In the upper panel, showing TNF-α mRNA findings, results are given as mean ± standard deviation. ^§^*P *< 0.1. Lower panel: gel showing the effect of temperature on myocardial expression of TNF-α mRNA; results are representative of six independent experiments in each group. **(b) **In the upper panel, showing TNF-α protein concentrations, results are given as mean ± standard deviation. *P < 0.05, ^§^*P *< 0.1. Lower panel: gel showing the effect of temperature on myocardial expression of TNF-α, as detected by Western blot. Band intensities for TNF-α were normalized for band intensities for actin. Results are representative of six independent experiments in each group (28°C = hypothermia, 37°C = normothermia). bp, base pairs; CPB cardiopulmonary bypass; M, molecular weight markers; TNF, tumour necrosis factor.

There was no TNF-α expression before institution of CPB; however, there was expression 30 min after establishing CPB and before cross-clamping of the aorta. At that time, both TNF-α gene expression and TNF-α concentrations tended to be lower in pigs operated on under moderate hypothermia than in the other animals (Figure 1a,b). Six hours after CPB, concentrations of TNF-α were lower in animals operated on under moderate hypothermia than in the others (*P *< 0.05; Figure 1a,b).

### Activation of nuclear factor-κB

**Figure 2:**
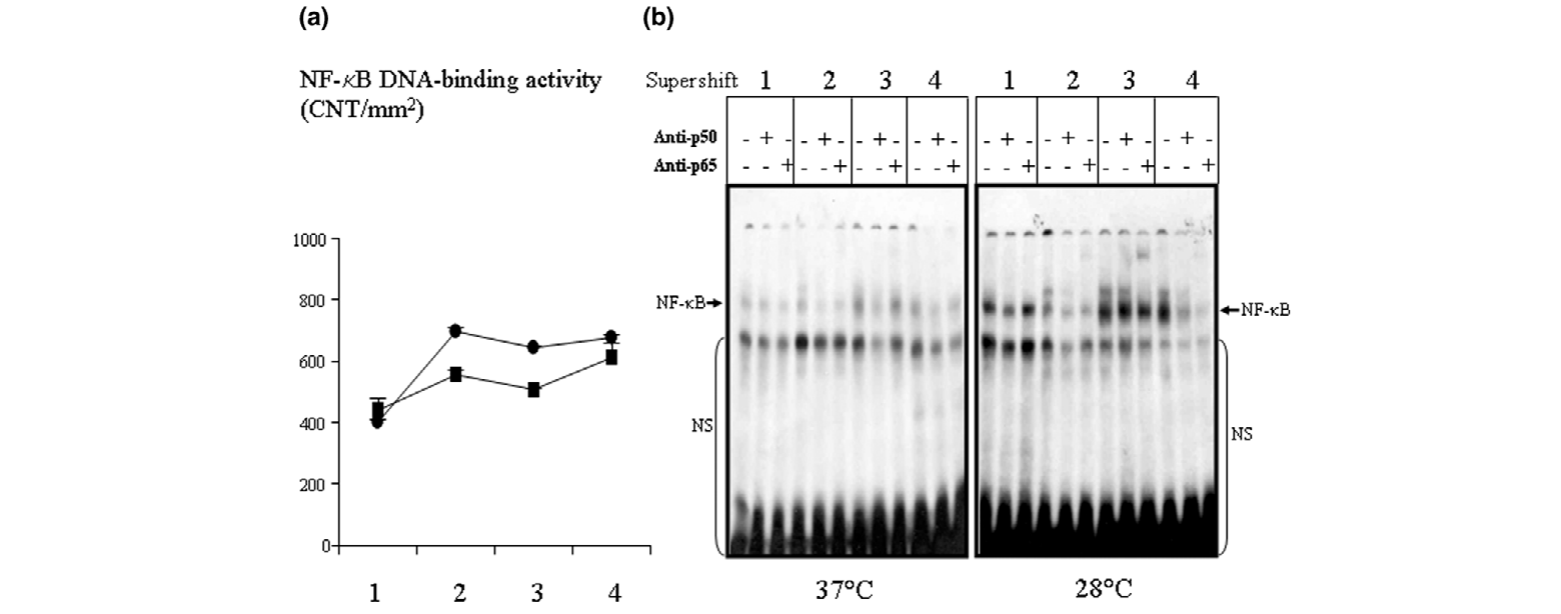
DNA binding activity of NF-κB: hypothermia versus normothermia. **(a) **DNA binding activity of NF-κB, as measured by EMSA and confirmed by supershift in myocardium, in pigs operated on under moderate hypothermia (28°C; circles) or normothermia (37°C; squares). The time points of evaluation were as follows: 1 = before CPB; 2 = before aortic cross clamping; 3 = before removal of aortic clamp; and 4 = 6 hours after CPB. Data are expressed as mean ± standard deviation. **(b) **Example gel showing the effect of temperature during CPB on activation of NF-κB as detected by EMSA and confirmed by supershift with anti-p50 and anti-p65 in pigs operated on under hypothermia (28°C) or normothermia (37°C). Results are representative of four independent experiments in each group. CNT, counts/mm^2^; CPB, cardiopulmonary bypass; EMSA, electrophoretic mobility shift assay; NF-κB, nuclear factor-κB; NS, nonspecific.

There was weak DNA binding activity of NF-κB before CPB that increased during and after CPB similarly in both animal groups (Table 2). The supershift experiment showed that the NF-κB DNA binding complex containing p50 and p65 was already present before CPB (Figure 2a,b). Phosphorylation of IκB-α in the myocardium paralleled DNA binding activity of NF-κB before, during and after CPB in both animal groups (Table 2).

### Phosphorylation of p38 mitogen-activated protein kinase

**Figure 3:**
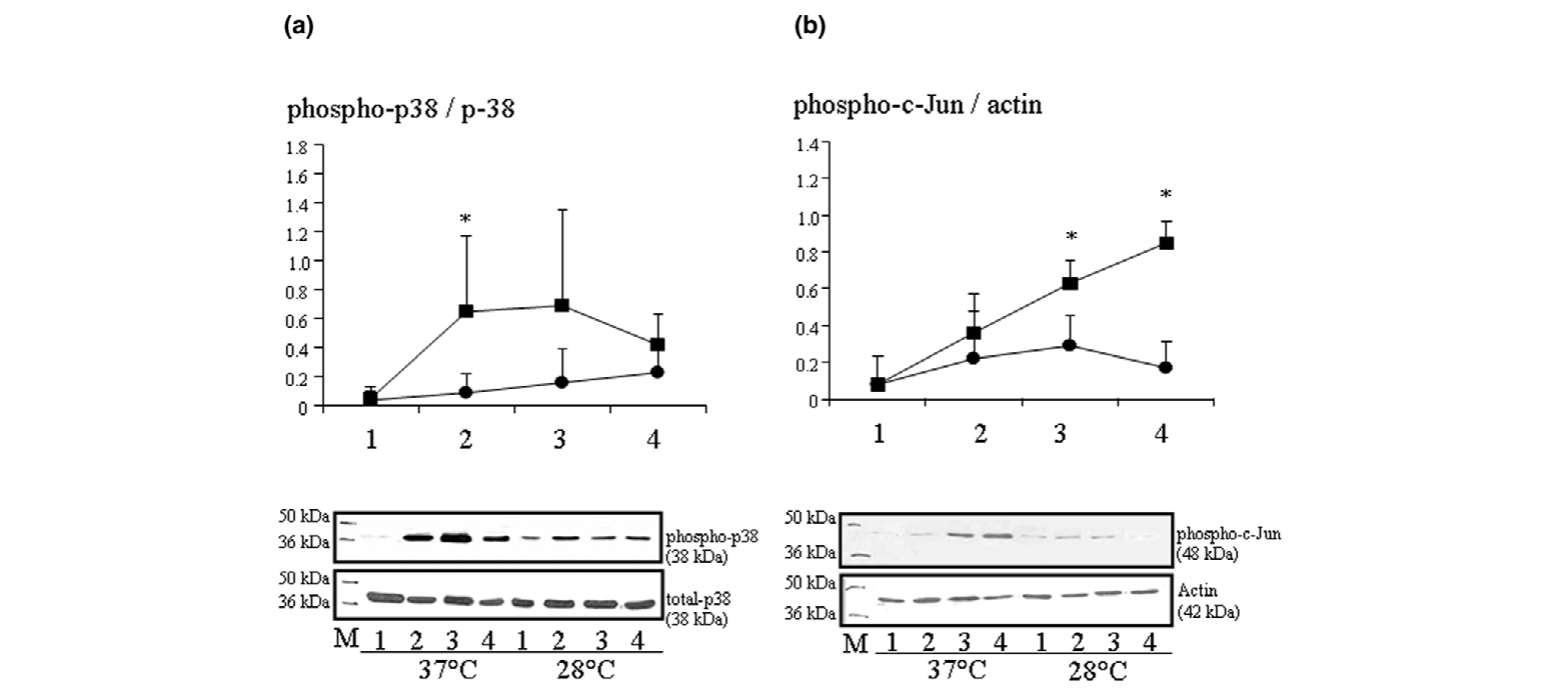
Activation of p38 MAPK and c-Jun: hypothermia versus normothermia. Shown is activation of p38 MAPK (phospho-p38 MAPK) and c-Jun (phospho-c-Jun) in myocardium of pigs operated on under moderate hypothermia (28°C; circles) or under normothermia (37°C; squares). The time points of evaluation were as follows: 1 = before CPB; 2 = before aortic cross clamping; 3 = before removal of aortic clamp; and 4 = 6 hours after CPB. **(a) **In the upper panel, showing phospho-p38 MAPK findings, results are given as mean ± standard deviation. *P < 0.05. Band intensities for phospho-p38 MAPK were normalized for band intensities of total p38 MAPK. Lower panel: gel showing the effect of temperature on myocardial expression of phospho-p38 MAPK detected by Western blot. Results are representative of six independent experiments in each group. **(b) **In the upper panel, showing phospho-c-Jun findings, results are given as mean ± standard deviation. *P < 0.02. Band intensities for phospho-c-Jun were normalized for band intensities of actin. Lower panel: gel showing the effect of temperature on expression of phospho-c-Jun in the nuclear extract detected by Western blot. Results are representative of four independent experiments in each group (28°C = moderate hypothermia; 37°C = normothermia). CPB, cardiopulmonary bypass; MAPK, mitogen-activated protein kinase.

There was weak phosphorylation of p38 MAPK before CPB in all animals. In those operated on under hypothermia, levels of phospho-p38 MAPK were lower during and after CPB than in animals operated on under normothermic conditions. In the latter animals, levels of phospho-p38 MAPK increased as soon as 30 minutes after CPB was established and reached a peak value just before removal of the aortic clamp (1 hour after ischaemia), and then decreased by 6 hours after CPB. Animals operated on under hypothermia exhibited lower levels of phospho-p38 MAPK 30 minutes after establishing CPB as compared with the other animals (*P *< 0.05; Figure 3a). Levels of phospho-c-Jun detected in the nuclear extract paralleled the activation of p38 MAPK in all animals during CPB, but continued to increase after CPB exclusively in those subjected to normothermia. In the other animals, levels of phospho-c-Jun were lower after removal of the aortic clamp and 6 hours after CPB (*P *< 0.05; Figure 3b).

### Activation of activating protein-1

**Figure 4:**
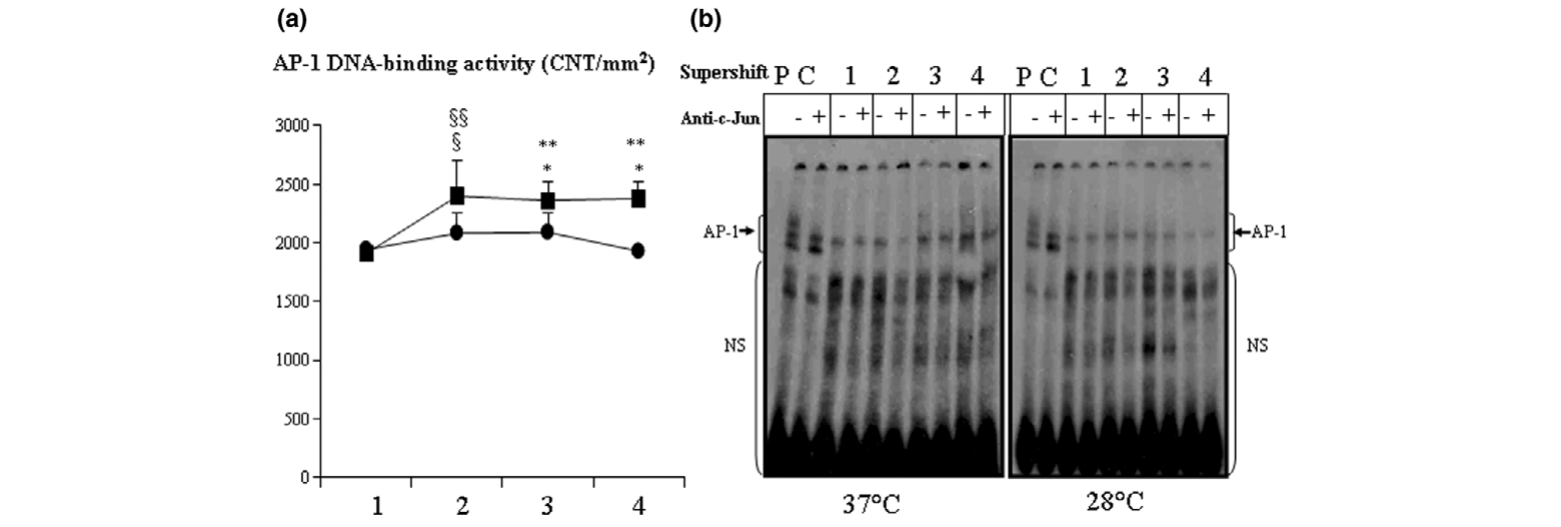
DNA binding activity of AP-1: hypothermia versus normothermia. **(a) **DNA-binding activity of AP-1, as measured by EMSA and confirmed by supershift in the myocardium, in pigs operated on under moderate hypothermia (28°C; circles) or normothermia (37°C; squares). The time points of evaluation were as follows: 1 = before CPB; 2 = before aortic cross clamping; 3 = before removal of aortic clamp; and 4 = 6 hours after CPB. Data are expressed as mean ± standard deviation. ^§^*P *< 0.1, **P < 0.05, versus prebypass value in normothermia group; ^§^*P *< 0.1, *P < 0.05 between both groups. **(b) **Example gel showing the effect of temperature during CPB on activation of AP-1, as detected by EMSA and confirmed by supershift, with anti-c-Jun antibody in pigs operated on under hypothermia (28°C) or normothermia (37°C). Results are representative of four independent experiments in each group. AP, activating protein; CNT, count/mm^2^; CPB, cardiopulmonary bypass; EMSA, electrophoretic mobility shift assay; NS, nonspecific; PC, positive control.

There was weak DNA binding activity of AP-1 before CPB in all animals. In animals operated on under hypothermia, AP-1 activity was lower during and after CPB than in those operated on under normothermia. In these latter animals, the DNA binding activity of AP-1 increased as soon as 30 minutes after CPB was established, remaining elevated for up to 6 hours after CPB (Figure 4a). Animals operated on under hypothermia exhibited lower DNA binding activity of AP-1 during and after CPB as compared with the others (*P *< 0.05 and *P *< 0.005, respectively; Figure 4a). The supershift experiment revealed that the AP-1 DNA binding complex containing c-Jun was present before and persisted during and after CPB (Figure 4b).

### Synthesis of inducible nitric oxide synthase and cyclo-oxygenase-2

iNOS was not detected before but 30 minutes after establishing CPB in all animals. Its synthesis increased during and after CPB without any difference between groups (table 2).

COX-2 levels increased during CPB in all animals. At 30 minutes after establishing CPB the COX-2 levels in animals operated on under moderate hypothermia were significantly lower than in the other animals (*P *< 0.005; Table 2).

## Discussion

This study shows for the first time that the repression of TNF-α associated with application of moderate hypothermia during CPB is associated with inhibition of the p38 MAPK/AP-1 pathway but not that of the NF-κB pathway.

In previous studies we reported that animals operated on under moderate hypothermic CPB have lower plasma levels and lower myocardial concentrations of TNF-α as well as less organ damage than do animals operated on under normothermic conditions [2,20]. In the present study we found that the anti-inflammatory effects of moderate hypothermia with repression of TNF-α and of its secondary mediator COX-2 are present as early as 30 minutes after initiation of CPB. This observation is supported by other investigators [22], who reported repression of TNF-α by hypothermia during ischaemia and early reperfusion periods in the liver.

The main aim of this work was to identify the upstream mechanisms that regulate the expression of TNF-α and COX-2, levels of which appear to be reduced by hypothermia. We therefore investigated both of the main signalling pathways involved in the expression of inflammatory mediators, namely the NF-κB [11] and AP-1 [23] pathways, and observed a differential effect of hypothermia on those transcription factors. Indeed, although in our series hypothermia led to reductions in phosphorylation of p38 MAPK and AP-1 activation, it did not affect activation of NF-κB in the myocardium. This confirms our previous work showing that hypothermia does not inhibit NF-κB activation in liver [21].

The inhibitory effect of hypothermia on p38 MAPK that we report here is supported by experimental work conducted in rat fibroblasts that showed inhibition of Raf [24], a mitogen-stimulated protein kinase that is an important intermediate to p38 MAPK activation by cold stress [25]. Via inhibition of Raf, hypothermia suppresses the phosphorylation of p38 MAPK *in vitro*, thereby inhibiting phosphorylation of c-Jun and AP-1 activation [22].

Regulation of the activity of AP-1 occurs at two levels, depending on its concentrations and on the level of its phosphorylation [26]. In the present study, there was no difference in levels of c-Jun in nuclear extract between the groups, but animals operated on in hypothermia exhibited lower phosphorylation of c-Jun and lower DNA binding activity of AP-1 during and after CPB than did those operated on in normothermia. Therefore, we suggest that hypothermia during CPB inhibits phosphorylation of p38 MAPK, which in turn suppresses its nuclear targets, namely phosphorylation of c-Jun and activation of the transcription factor AP-1.

The clinical relevance of NF-κB and p38 MAPK/AP-1 activation in myocardium has been pointed out by several studies suggesting that both pathways are important actors in the failing heart [18,27]. NF-κB plays a central role in the myocardial inflammatory response, including TNF-α secretion by cardiomyocytes in response to systemic endotoxin [28]. However, NF-κB activation is also necessary for later preconditioning of the myocardium, and thus a potential role of this transcription factor for myocardial protection has been suggested [11]. The important role played by p38 MAPK/AP-1 in the myocardial inflammatory response is widely recognized [18,23]. Indeed, p38 MAPK activation is sufficient to induce inflammatory cytokine expression including TNF-α in cardiomyocytes *in vitro *and *in vivo *[18]. A recent study [18] showed that the inhibition of p38 MAPK activities blocks TNF-α secretion in transgenic hearts. The congruency of the effect of moderate hypothermia on phosphorylation of p38 MAPK and activity of AP-1, and on expression of TNF-α suggests that, in our model, moderate hypothermia during CPB represses the expression of TNF-α in the myocardium by selective inhibition of the p38 MAPK/AP-1 pathway.

In a similar *in vivo *model, we previously showed that hypothermia during CPB confers myocardial protection by inhibiting intramyocardial expression of TNF-α [2,20]. Cardiodepressive effects of TNF-α have been ascribed to immediate negative inotropic effects on cardiomyocytes mediated by sphyngosine, independent of NO, and to delayed NO-dependent negative inotropic effects [29]. High local concentrations of NO related to induction of iNOS are associated with myocardial cell damage and cell death [30]. In the present study, hypothermia during CPB did not inhibit synthesis of iNOS in myocardium despite repression of TNF-α. That expression of iNOS in cultured cardiac myocytes does not increase in response to TNF-α and that TNF-α does not influence iNOS mRNA expression and NO release in the isolated rat heart [31-33] supports our observation. In addition, endotoxin-induced TNF-α depresses myocardial contractility of isolated rat hearts independent of NO synthesis [34].

COX-2 is upregulated by various factors, including TNF-α, via the transcription factors NF-κB and AP-1 [11,35]. The latter is the major promotor element involved in COX-2 expression in cardiomyocytes [36]. Significant expression of COX-2 has been demonstrated in the myocardium of patients with congestive heart failure and in rat heart after treatment with endotoxin [37,38]. In our series expression of COX-2 paralleled the expression of TNF-α and activation of AP-1 during and after CPB, suggesting that COX-2 expression was mediated by TNF-α through AP-1. Myocardial depression in isolated rat hearts in response to staphylococcal α-toxin results from COX-2 derived thromboxane A_2 _liberation, leading to coronary vasconstriction and perfusion mismatch [39,40]. In the present study, animals operated on under hypothermia did not differ from the other animals with respect to haemodynamics, but the fact that they had lower levels of lactate than did the pigs operated on under normothermia indicates that they had better tissue perfusion.

## Conclusion

Our findings suggest that myocardial repression of TNF-α and COX-2 related to moderate hypothermia during CPB is due to inhibition of the p38 MAPK/AP-1 pathway but not that of NF-κB.

## Key messages

• 	Hypothermia during cardiac surgery reduces myocardial inflammation

• 	The anti-inflammatory effect of hypothermia relates to repression of TNF-α and COX-2

• 	Hypothermia inhibits the p38/AP-1 pathway but not NF-κB

• 	Mitigating the inflammatory response to cardiac surgery might improve postoperative outcome

## Abbreviations

AP = activating protein; COX = cyclo-oxygenase; CPB = cardiopulmonary bypass; IκB = NF-κB inhibitory protein; iNOS = inducible nitric oxide synthase; MAPK = mitogen-activated protein kinase; NF-κB = nuclear factor-κB; NO = nitric oxide; RT-PCR = reverse transcriptase polymerase chain reaction; TNF = tumour necrosis factor.

## Competing interests

The authors declare that they have no competing interests.

## Authors' contributions

M-CS and MQ designed the study. JFV-J, MQ, KS and MS were responsible for data acquisition. MQ and KS performed analyses of gene and protein expression. MQ and MW performed analyses and interpretation of DNA binding activity. RM performed statistical analyses. MQ and M-CS drafted the manuscript. All authors read and approved the final manuscript.

## Figures and Tables

**Table 1:** Oesophageal and myocardial temperatures before, during, and after CPB in pigs operated on under moderate hypothermia or normothermia

Time point	Esophageal temperature			Myocardial temperature		
	
	Hypothermia (28°C)	Normothermia (37°C)	*P*	Hypothermia (28°C)	Normothermia (37°C)	*P*
Before CPB	35.9 ± 0.5	36.1 ± 0.9	NS	35.8 ± 0.6	35.7 ± 1.5	NS
10 minutes after institution of CPB	30.1 ± 4.4	36.7 ± 0.4	0.01	28.2 ± 0.4	36.4 ± 1.0	0.001
10 minutes after aortic cross clamping	28.1 ± 0.09	36.6 ± 0.6	0.001	17.2 ± 3.4	17.4 ± 2.4	NS
60 minutes after aortic cross clamping	33.3 ± 1.7	36.2 ± 0.3	0.05	28.1 ± 2.5	31.5 ± 1.5	NS
End of CPB	35.0 ± 1.9	36.6 ± 0.3	NS	34.8 ± 0.5	35.9 ± 1.0	NS
6 hours after CPB	36.1 ± 0.8	38.0 ± 0.5	0.005	36.4 ± 1.1	38.6 ± 0.6	NS

Results are expressed as mean ± standard deviation. CPB, cardiopulmonary bypass; NS, not significant.

**Table 2:** Intramyocardial DNA binding activity of NF-κB and synthesis of inflammatory mediators before, during, and after CPB in pigs operated on under moderate hypothermia or normothermia

Parameter	Before CPB		Before aortic cross clamping		Before removal of aortic clamp		6 hours after CPB	
	
	28°C	37°C	28°C	37°C	28°C	37°C	28°C	37°C
Activity of NF-κB (count/mm^2^)	400.8 ± 14.3	439.2 ± 63.3	696.4 ± 25.9	555.1 ± 32.1	643.5 ± 7.45	507.3 ± 9.22	676.3 ± 12.8	611.0 ± 98.7
Phospho-IκB-α	0.18 ± 0.32	0.24 ± 0.45	0.71 ± 1.13	0.70 ± 0.87	0.72 ± 0.96	0.64 ± 0.49	0.85 ± 0.59	0.66 ± 0.43
iNOS	0.00 ± 0.00	0.00 ± 0.00	0.47 ± 0.54	0.29 ± 0.55	0.58 ± 0.97	0.48 ± 0.40	0.88 ± 0.58	0.47 ± 0.48
COX-2	0.09 ± 0.1	0.11 ± 0.08	0.16 ± 0.09	0.50 ± 0.18*	0.54 ± 0.06	0.68 ± 0.33	0.25 ± 0.26	0.47 ± 0.13

Results (mean ± standard deviation) are shown as the ratio of protein levels of phospho-IκB-α, iNOS and COX-2 to actin. *P < 0.05 between groups. COX, cyclooxygenase-2; CPB, cardiopulmonary bypass; iNOS, inducible nitric oxide synthase; NF-κB, nuclear factor-κB; phospho-IκB-α, phosphorylation of inhibitor of NF-κB α.
